# False Positive COVID-19 Antibody Test in a Case of Granulomatosis With Polyangiitis

**DOI:** 10.3389/fmed.2020.00399

**Published:** 2020-07-07

**Authors:** Argyrios Tzouvelekis, Theodoros Karampitsakos, Anastasia Krompa, Evangelos Markozannes, Demosthenes Bouros

**Affiliations:** First Academic Department of Respiratory Medicine, National and Kapodistrian University of Athens, Athens, Greece

**Keywords:** COVID-19, granulomatosis with polyangiitis, false positive, cross-reactivity, antibodies

## Abstract

Collateral damage due to 2019 novel coronavirus disease (COVID-19) represents an emerging issue. Symptoms of COVID-19 are not disease-specific. Differential diagnosis is challenging and the exclusion of other life-threatening diseases has major caveats. In the era of this pandemic, diagnosis of other life-threatening diseases might delay treatment. The Food and Drug Administration has recently authorized the first antibody-based test for COVID-19; however, RT-PCR of nasopharyngeal or oropharyngeal swabs remains the recommended test for diagnosis. We present the first report of a false positive COVID-19 antibody test in a case of Granulomatosis with Polyangiitis (GPA). Specifically, the case concerns an 82-year-old female, never smoker, who was admitted to our hospital with symptoms of fever and general fatigue that had lasted 7 days. She already had a positive IgM test for COVID-19, yet multiple RT-PCR tests had returned as negative for SARS-CoV-2. In the following days, her renal function deteriorated, while hematuria and proteinuria with active urinary sediment developed. Based on high clinical suspicion for ANCA-associated vasculitis, we performed a complete immunologic profile which revealed positive c-ANCA with elevated titers of anti-PR3. Pulses of methylprednisolone along with cyclophosphamide were applied. At day 10, treatment response was noticed as indicated by respiratory and renal function improvement. This report highlights the need for meticulous patient evaluation in order to avoid misdiagnosis in the era of COVID-19.

## Introduction

The emergence and spread of 2019 novel coronavirus disease (COVID-19), as well as the associated acute respiratory distress syndrome (ARDS), are causing a growing global public health crisis (1). Symptoms of COVID-19 are not disease-specific. Thus, differential diagnosis and exclusion of other life-threatening diseases could be challenging. Collection of an upper respiratory nasopharyngeal (or oropharyngeal) swab and evaluation through real-time reverse transcriptase polymerase chain reaction (RT-PCR) is currently recommended for initial COVID-19 testing (1). The Food and Drug Administration has recently authorized the first antibody-based test for COVID-19. However, cross-reactivity and diagnostic accuracy of antibody-based tests is currently a matter of investigation (2–4). Our aim is to present the first report of a false positive COVID-19 antibody test in a case of Granulomatosis with Polyangiitis (GPA).

## Case Report

**Figure 1:**
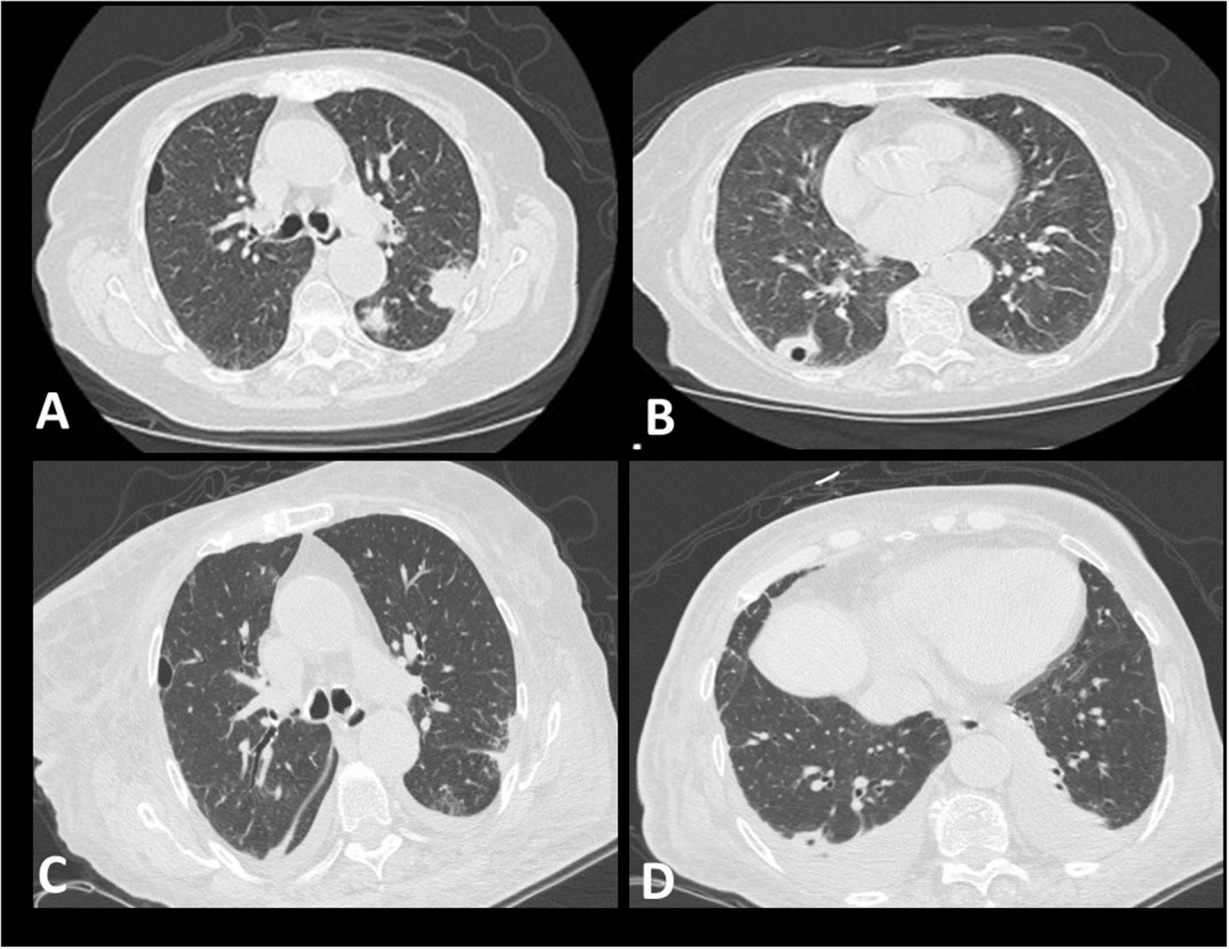
Radiological features. **(A,B)** showing multifocal consolidative opacities in the left upper lobe and one cavitary lesion in the right lower lobe **(A,B)** (day 1). Subtle ground glass opacities across the bronchovascular bundle can also be seen **(B)**. Mild improvement of the radiographic appearance of the lesions is evident following treatment with pulses of methylprednisolone and cyclophosphamide **(C)**. Small bilateral pleural effusions are shown, indicating possible fluid overload **(D)**.

An 82-year-old female, non-smoker, with a history of arterial hypertension, was admitted to our hospital with symptoms of fever and general fatigue that had lasted 7 days. She had a positive IgM test for COVID-19 (Anachem Diagnostics-Ref B251C) prior admission. On admission, she was febrile (Θ°C = 37.8°C), hemodynamically stable (BP = 130/60 mm Hg, HR= 88 bpm), and her oxygen saturation was 97% (FiO_2_: 21%). She was alert and awake, with no signs of respiratory distress. Lung auscultation did not reveal abnormal sounds. Laboratory tests showed normocytic, normochromic anemia (Ht = 28.2%), leukocytosis (white blood cells = 16.12 K/μl), high levels of C-Reactive Protein (CRP = 29.91 mg/dl), and mild renal impairment (urea = 61 mg/dl, creatinine = 1.3 mg/dl). High Resolution Chest Computed Tomography (HRCT) depicted multifocal consolidative opacities, including one cavitary lesion in the right lower lobe. The cavitary lesion was initially considered as an air-bubble sign, a sign previously described in patients with COVID-19 infection (1). Subtle areas of ground glass opacities across the bronchovascular bundle in both lower lobes were also noticed (Figures 1A,B). Treatment with hydroxychloroquine 200 mg thrice a day, ceftriaxone 2 g once daily, and azithromycin 500 mg once daily was commenced. An upper respiratory nasopharyngeal swab sample was obtained at day 1 and an RT-PCR test was negative for SARS-CoV-2. Over the following 2 days, her renal function further deteriorated (creatinine = 2.0 mg/dl), while hematuria and proteinuria with active urinary sediment developed. The patient progressed to respiratory failure as indicated by SaO_2_ = 94%, FiO_2_: 36%. Two more nasopharyngeal samples were obtained, and RT-PCR tests returned as negative for SARS-CoV-2. Based on high clinical suspicion for ANCA-associated vasculitis, we performed a complete immunologic profile which revealed positive c-ANCA (immunofluorescence) with elevated titers of anti-PR3 (300 IU), at day 4. Laboratory tests for other pathogens, including Influenza A and B, Streptococcus pneumoniae, and Legionella, were negative. Procalcitonin levels were mildly elevated (procalcitonin = 0.41 ng/ml). Based on a compatible radiological and laboratory pattern, the diagnosis of GPA was set. Pulses of methylprednisolone for three days (1 g per day), along with cyclophosphamide (1 g), were applied. Despite appropriate treatment, only minor radiological improvements were noticed, while oxygenation and renal function continued to deteriorate (SaO_2_ = 94% with FiO_2_: 60% compared to SaO_2_ = 94% with FiO_2_: 36%, blood creatinine levels = 2.0 compared to 3.1) (day 8). Treatment with diuretics commenced, due to the development of a pulmonary edema. Two more pulses (1 g) of methylprednisolone were applied, followed by maintenance doses of 1 mg/kg. At day 10, treatment response was noticed as indicated by respiratory and renal function improvement (SaO_2_ = 98% with FiO_2_: 36%, reduction in blood creatinine levels 2.4). During the following days, the patient remained clinically stable under a maintenance dose of corticosteroids. Post-treatment HRCT depicted mild improvement of the radiographic appearance of the lesions. Small bilateral pleural effusions were shown, indicating possible fluid overload (Figures 1C,D).

## Discussion

Our report is the first case of PR3—ANCA positive vasculitis leading to a false positive COVID-19 antibody test, potentially due to cross-reactivity. The concept of COVID-19 induced vasculitis in the context of viral-induced ANCA-associated vasculitis could not be verified as three nasopharyngeal swab tests were negative for SARS-CoV-2 (2). Slight elevations of procalcitonin levels may also be attributed to the systemic vasculitis as has been previously reported (5).

The antibody test (Anachem Diagnostics-Ref B251C) used in the study had a reported sensitivity and specificity of 92 and 99.5% for COVID-19, respectively. ANCA positivity has been associated with the presence of other false positive antibodies due to cross-reactivity; yet, there were no reports for cross reactivity with SARS-CoV-2 (3, 6–8). Interestingly, cross-reaction of the previous coronavirus (SARS-CoV) antigen with autoantibodies in autoimmune diseases had already been reported (9). It has been suggested that patients with autoimmune diseases, including ANCA-positive-vasculitides, present with a plethora of autoantibodies to cell antigens, and SARS-CoV Vero E6 cell lysates used as antigens could have led to such false-positive reactions (9).

This report highlights the importance of thoughtful evaluation of COVID-19 antibody tests in clinical practice, especially in patients with autoimmune diseases. Although this is a typical GPA case, this report shows the danger of delay in GPA diagnosis in the case where a clinician is basing diagnosis on the positive antibody test for COVID-19. While IgG testing might be used to identify re-convalescent patients, the clinical utility of antibody testing in the acute symptomatic phase is unclear and thus meticulous evaluation is needed to avoid erroneous interpretations for positive IgM tests. A limitation of this report is that it represents a single case. Therefore, further reports are needed to verify the frequency of this phenomenon. To this end, antibody tests should be well-studied prior entering widely into clinical practice; most importantly, though, is that clinicians should be vigilant and interpret results based on the pre-test clinical probability. If used appropriately, antibody tests could be game changers for COVID-19 suppression. If not, similar cases may be encountered, leading to fatal collateral damage (10, 11).

## Data Availability Statement

The raw data supporting the conclusions of this article will be made available by the authors, without undue reservation.

## Ethics Statement

Written informed consent was obtained from the individual(s) for the publication of any potentially identifiable images or data included in this article. This report has been approved by the Institutional Review Board.

## Author Contributions

AT, AK, and EM mainly cared for the patient. AT and TK wrote and edited the paper. DB revised the paper for important intellectual content. All authors read and approved the final version of the paper.

## Conflict of Interest

The authors declare that the research was conducted in the absence of any commercial or financial relationships that could be construed as a potential conflict of interest.
